# Collaboration process for integrated social and health care strategy implementation

**DOI:** 10.5334/ijic.816

**Published:** 2012-05-18

**Authors:** Jukka Korpela, Kalle Elfvengren, Tanja Kaarna, Merja Tepponen, Markku Tuominen

**Affiliations:** Roce Partners, Tekniikantie 14, FI-02150 Espoo, Finland; Department of Industrial Management, Lappeenranta University of Technology, P.O. Box 20, 53851 Lappeenranta, Finland; South Karelia District of Social and Health Services, P.O. Box 24, 53101 Lappeenranta, Finland; South Karelia District of Social and Health Services, P.O. Box 24, 53101 Lappeenranta, Finland; Department of Industrial Management, Lappeenranta University of Technology, P.O. Box 20, 53851 Lappeenranta, Finland

**Keywords:** Integrated social and health care, group decision support system, GDSS, strategic planning, strategy implementation, collaboration

## Abstract

**Objective:**

To present a collaboration process for creating a roadmap for the implementation of a strategy for integrated health and social care. The developed collaboration process includes multiple phases and uses electronic group decision support system technology (GDSS).

**Method:**

A case study done in the South Karelia District of Social and Health Services in Finland during 2010–2011. An expert panel of 13 participants was used in the planning process of the strategy implementation. The participants were interviewed and observed during the case study.

**Results:**

As a practical result, a roadmap for integrated health and social care strategy implementation has been developed. The strategic roadmap includes detailed plans of several projects which are needed for successful integration strategy implementation. As an academic result, a collaboration process to create such a roadmap has been developed.

**Conclusions:**

The collaboration process and technology seem to suit the planning process well. The participants of the meetings were satisfied with the collaboration process and the GDSS technology. The strategic roadmap was accepted by the participants, which indicates satisfaction with the developed process.

## Introduction

The South Karelia District of Social and Health Services (Eksote) executes a challenging and complex health care integration process. The goal of the newly established organisation is to ensure equal access to social and health care services to all citizens in the region, across the boundaries of municipalities. The effectiveness of service delivery has been enhanced due to better co-operation of different social and health care organisations.

The integration of care is a fundamental challenge in the field of social and health care. Developing strategies and operating models that enable the functioning of client-oriented integrated social and health care within an integrated organisational structure, as well as ensuring effective change management, require tools. Eksote is a huge, exceptional structural solution where many different organisations have been merged into one organisation. To get this type of a new organisation functioning successfully to achieve its objectives, strong and effective change management and appropriate methods to execute it are required.

This paper presents a case study of implementing an integrated health care strategy at Eksote. The paper introduces a multi-phase collaboration process for developing commonly agreed tasks to implement the strategy in the new organisation. These tasks can be described as a strategic roadmap for the organisation. Three of the authors of this study have been closely involved in the development of the Eksote integration (the development director of Eksote, the project manager and the integration consultant), and two of the authors are university researchers specialized in collaboration processes and technologies.

## Integrated social and health care services at Eksote

The provision of integrated health and social care has been advocated by policy-makers in Europe [1–2]. Integrated care is a frequently used concept, which has so far not been unambiguously defined [3]. At the moment various kinds of definitions are used [4]. The different definitions have emphasised the perspectives of the client, service structure, or the financier, mainly the society. For example in the Procare project the term ‘integrated care’ stood for care services which were coordinated by one unit aiming to ensure cost effectiveness, better quality and increased client satisfaction [5].

The purpose of integrated care is to provide care without a service gap, fragmentation or lack of cooperation [6]. The main objectives from the perspective of the society are cost effectiveness and enhancing productivity [5, 7]. Other objectives include furthering the use of services that are based on the needs of clients, and decreasing the use of expensive hospital services [8].

Alaszewski et al. [9] identify structural integration as one approach to integrated care. Structural integration is mainly a matter of organisational structure (e.g. bringing together the staff and resources in a single organisation under a unified hierarchical structure). Their study suggests that structural integration may improve the quality of homecare, as it creates favourable conditions for deeper integration and removes obstacles hindering interdisciplinary collaboration. However, in order to be successful, strong change management is essential [10].

**Figure 1:**
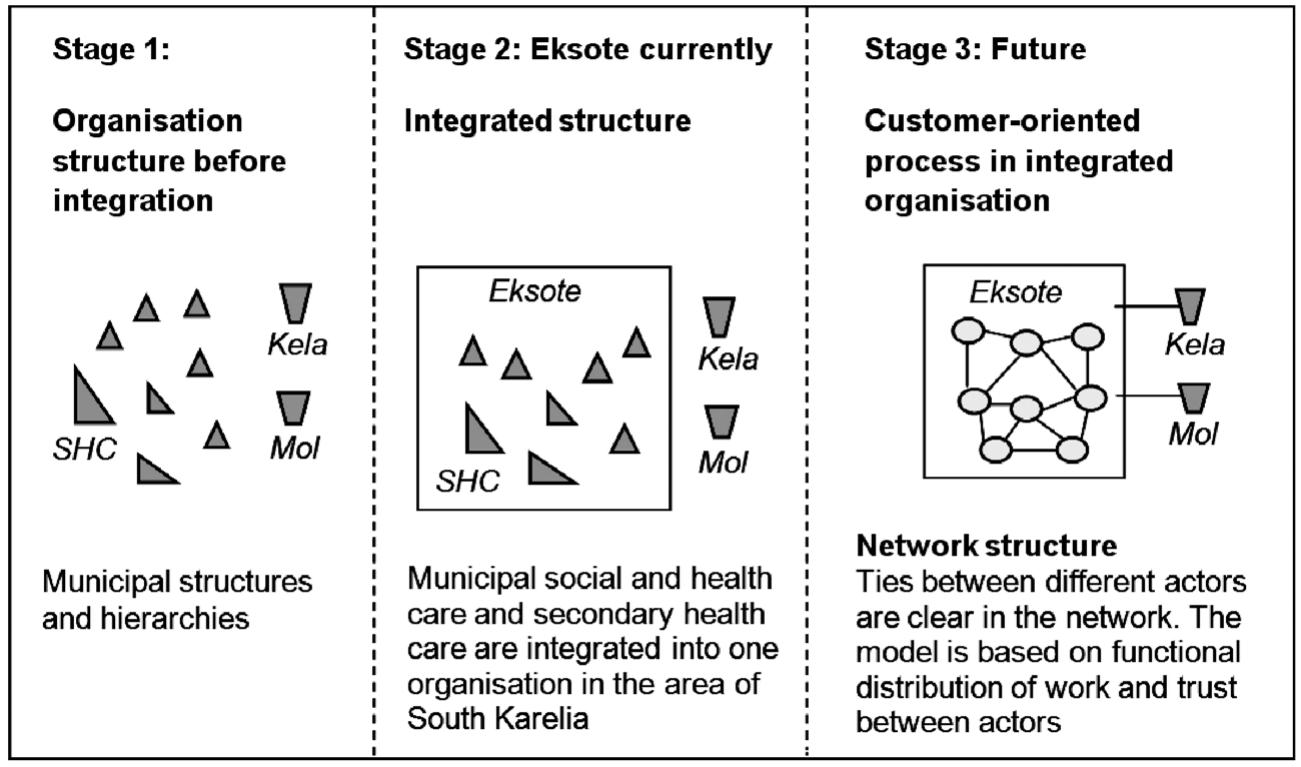
Development of organisational structure at Eksote.

In South Karelia, a region situated in South-Eastern Finland, the previously separate municipal health care and social services (see Figure 1, stage 1) were integrated into a new organisation called Eksote in the beginning of 2010 (Figure 1, stage 2). Since then Eksote has arranged secondary health care, primary health care, care for the elderly, and social welfare services for its eight member municipalities. Eksote works for delivering patient-oriented care to the approximately 130,000 citizens of South Karelia. It employs approximately 4100 people and has a budget of 370 million euros. Eksote operates in a geographical area of over 5600 square kilometres. The main reasons for the large organisational change, where Eksote was established, were solving claimed problems in economy, efficiency and service quality (e.g. equal access, continuity, client-orientation, and need-based service).

There is a dual structure in health care in Finland: one for primary care with municipality-based units and the other for secondary care with region-based units. Two types of organisations have provided primary care after the recent extensive mergers started in the 1990s, where municipal primary care and social service organisations were merged (e.g. [10]). These two types of organisations for primary care are (1) primary health care centres and (2) joint health and social centres. An exceptional feature of the Finnish primary care system has always been an extensive array of services, which grew enormously when all social services were included under the same administrative roof with primary care. Finnish municipalities are responsible for organising primary health care.

The other tier of the Finnish health care system is region-based secondary health care (SHC), which was provided by 21 specialised care districts maintained by 360 municipalities until 2009, when one of these 21 districts (South Karelia) was combined to Eksote. Since then the remaining 20 specialised care districts have continued to provide only secondary health care. The strict primary-secondary care divide existed in the whole of Finland until the establishment of Eksote.

Both systems (primary and secondary health) have extensive public sector administration with local politicians as decision-makers at the top with hierarchical management structures under them. Eksote is a public sector organisation.

The integrated organisation creates excellent possibilities for developing social and health care services for a larger area as a single entity. Eksote plans to develop the integrated service structure further into the next stage (Figure 1, stage 3), where the connections and ties between different actors within the organisation are clear and functional. Additionally, Eksote hopes to create a partnership network and to strengthen flexible and client-oriented cooperation with different actors, such as the Social Insurance Institution of Finland (Kela), the Employment and Economic Development Office (Mol), the third sector, the private sector, and other municipal actors. Integrated service processes are more functional, cost effective and client-oriented.

The goal of Eksote is to increase the productivity of work by developing the processes without decreasing the quality of care. Eksote aims to define a new process model based on “a clean slate”, so that the old municipal or organisational borders will not affect the planning process. The development team^1^ at Eksote is currently working to create new client-oriented and cost-effective service processes that span over different professional areas.

The traditional way of thinking, stemming from units and municipalities, has to be replaced with comprehensive client and process-oriented thinking in order to ensure that the strategy of Eksote will succeed. The new organisation has to create commonly agreed operating models which can steer all actors towards the common goal. In the former, traditional model, all social and health care actors functioned in different organisations, and furthermore, the services were arranged by eight different municipalities separately (Figure 1, stage 1). Secondary health care (SHC) was a region-based multipurpose organisation responsible for health and social services for the whole area before Eksote was formed. Thus, before the merger to Eksote, SHC was of a very different form, including the organisations of health, social service, and joint health and social service units.

Figuratively speaking, Eksote turns territories into resources. One of the most essential functional objectives is to reduce the use of institutional care radically. The whole care process of the client has to be seen as one continuum which begins from home and continues fluently back home again. This requires seamless cooperation in the network of actors. Implementing the strategy requires turning the strategic goals into distinct practical actions. Finding sufficient time for working on the strategic goals and new common operating models was found challenging in the new organisation. Those in charge were well aware of the development needs of the process in their own specialty areas. The development needs were often connected to the interfaces between the previously separated organisational units. A method enabling the collecting of executive and middle management level managers’ insights and allowing working on ideas was called for.

## GDSS technology to support collaboration

In complex planning and decision-making situations which influence many organisational units and other actors, it is important to create consensus and commitment to the decisions among the people who are needed in successful implementation of the plans. Even if the decision or plan seems good on paper, the implementation process could be a total failure if the people in the organisation are not satisfied and willing to execute it in practice. The commitment and approval of the people are in a very important role in successful implementation of the decisions. If the people can participate enough in the planning process, the reasons affecting the final plan become clearer to them. When the people understand the background of the problem better, they can plan the possible actions and understand the whole situation better. When their understanding is on a higher level, they can approve of the decisions and are committed to them [11].

A group decision support system (GDSS) is a collection of software applications aimed at facilitating group work, allowing collaboration either on-site or out of different places. A typical face-to-face GDSS facility comprises a variable number of terminals in a network, combined with various audiovisual systems. The concept aims at bringing systematic procedures and benefits from IT development to support group work in a manageable way. This is done by enhancing the process gains and reducing the process losses occurring in a teamwork environment.

Collaboration technologies support the group work process and improve the productivity of meetings, either by speeding up the meeting process or by improving the quality of the results. The GDSS is superior to a conventional meeting. It offers several possibilities for supporting a group in promoting cooperation and effectiveness (see Table 1).

**Figure 2:**
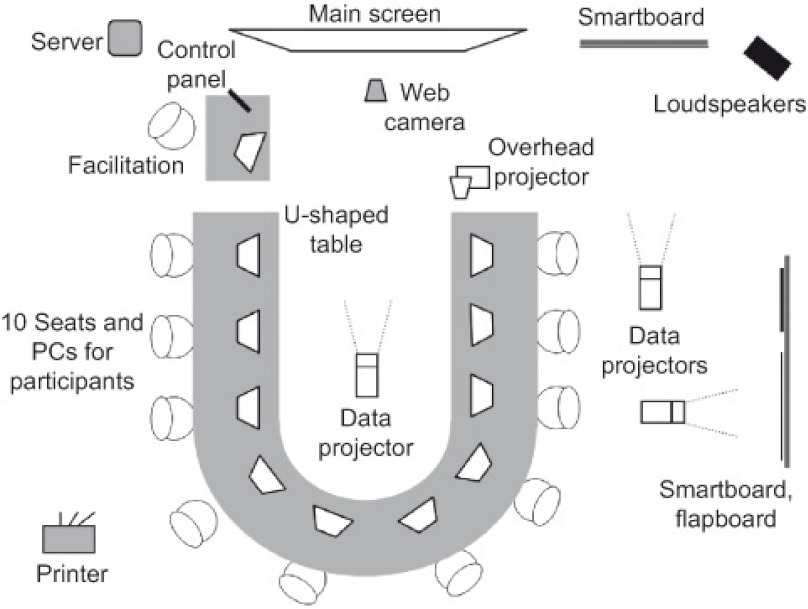
Overview of the GDSS facility.

The decision room at Lappeenranta University of Technology was utilized in the Eksote case. The room is a PC-equipped Local Area Network-based meeting room designed especially for decision-making, and various commonly used decision support software have been installed. The main group support software of the studio is the GroupSystems (www.groupsystems.com). The GroupSystems comprises half a dozen different tightly integrated applications (e.g. Categorizer and Vote), which support different phases of group processes, such as brainstorming, list building, information gathering, voting, organising, prioritising, and consensus building. Figure 2 illustrates the layout of the facility.

The laboratory has been designed to look like an ordinary meeting room, but the horseshoe-shaped conference table houses ten workstations hidden inside the table, which allows quick and flexible switching between computer-supported and ordinary meeting activities. In addition, the displays are under the glass surface of the table so that the displays do not dominate the appearance of the room, and every participant has a direct eye contact with the others.

## Framework for applying GDSS at Eksote

**Figure 3:**
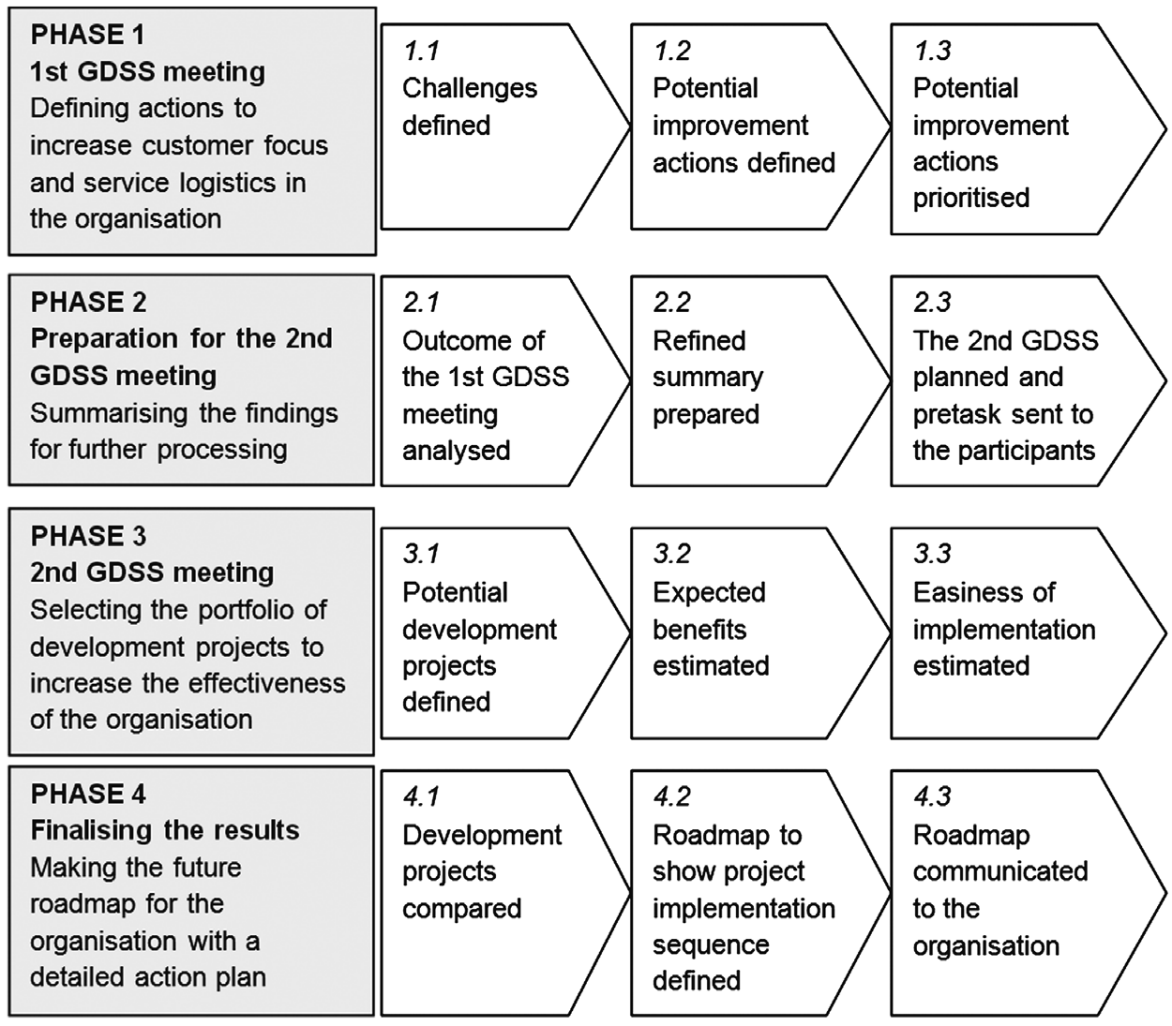
Framework for the strategy planning process.

The main target of the GDSS-supported collaboration process was to create a detailed roadmap to successful integration strategy implementation. The collaboration process was carried out in a period of two months. The collaboration process was divided into four main phases, two of them supported by GDSS meetings, as shown in Figure 3.

An expert panel of 13 participants was used in the collaboration process. The participants represented different functions in the Eksote organisation, and most of them belonged to the management team. The participants were directors and managers, for example the development director, CEO, health care service manager, elderly-care service manager, family and social care service manager, chief administrative physician, mental health service manager, and director of homecare for the elderly.

### Phase 1

The objectives of phase 1 were to define the actions needed to increase customer focus and to improve service logistics in the organisation. The objectives for the first phase were chosen on the basis of the existing strategic targets of Eksote. The first GDSS meeting was structured to focus on identifying the current and future improvement needs in these two areas, on defining the actions necessitated by the improvement needs and on prioritising the defined actions. Thus, the targeted outcome of the first phase of the strategy planning process consisted of in-depth understanding of the improvement needs and improvement actions needed in customer focus and service logistics.

The structure of the first GDSS meeting is described in detail in Table 2. The meeting was started by a brief introduction to the GDSS approach and tools by the GDSS facilitator. The participants found the approach and tools easy to understand, and thus no actual hands-on training on the GroupSystems software was organised at this stage.

The meeting was structured into two main tasks. The first main task focused on increasing the customer focus, and it included three subtasks. The first subtask was to define the most important current challenges Eksote as an organisation was facing in the area of customer focus. The GroupSystems Categorizer software was used for collecting proposals for the challenges, and the GroupSystems Vote software was used for prioritising the identified challenges. After the challenges with the GroupSystems Categorizer had been collected, each challenge was discussed to ensure that everybody understood the meaning in a similar way. Furthermore, duplicates were removed and the challenges were divided into four main categories. The participants were also allowed to comment on any proposal by using the GroupSystems Categorizer during the discussion. The challenges were then prioritised within each category by using the GroupSystems Vote.

When the main challenges with regard to customer focus had been identified, the next subtask was to define potential actions to improve the situation. The GroupSystems Categorizer was used for collecting ideas, and discussion was used for grouping the ideas into main categories. The outcome was altogether 51 potential actions divided into three main categories. The last subtask with regard to customer focus was to prioritise the potential actions within the three main categories. The GroupSystems Vote with a voting scale from 1 to 10 was used, and the voting results were then jointly discussed to reinforce the consensus on the importance of the potential actions.

The second main task of the GDSS meeting was to define the actions for improving service logistics in the organisation. The approach was basically similar to the one used in the first main task, but due to lack of time the challenges were not defined by using the GroupSystems Categorizer but only gone through in a joint discussion. The discussion formed a good foundation for generating ideas about the potential actions to be taken to improve service logistics performance. Altogether 51 potential actions were identified, which were prioritised after a joint discussion by using the GroupSystems Vote.

### Phase 2

The output of the first GDSS meeting was very extensive. In order to ensure full benefit of the output, a four-member sub-team of the meeting participants summarised the findings for further processing. In this phase the outcome of the GDSS meeting was analysed thoroughly, a refined summary was prepared, and the second GDSS meeting was planned. Furthermore, a preparatory task for the second GDSS meeting was defined and sent to the participants.

**Figure 4:**
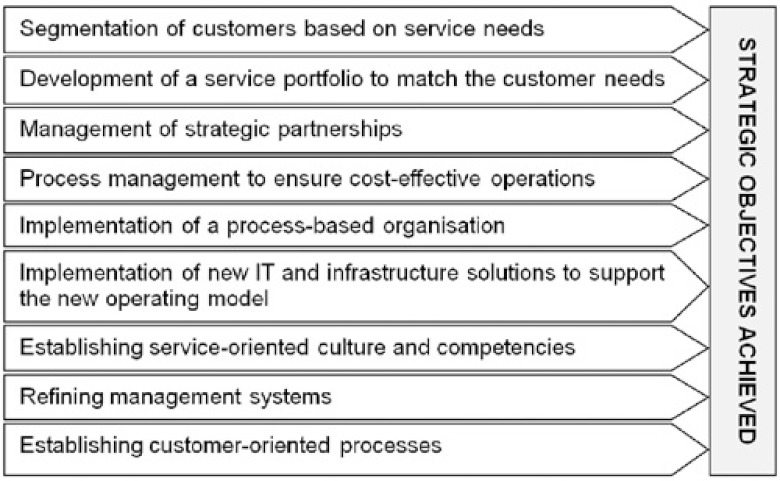
The focal development areas.

One task in the first GDSS meeting was to identify and prioritise the challenges Eksote was facing. Altogether 101 challenges were identified. The results were analysed after the meeting, and nine focal areas for development (see Figure 4) emerged. To summarise, the development needs concerned creating a service portfolio to match the customers’ needs, establishing a process-based way of working, improving service logistics, and utilising innovative IT solutions to enable the organisation to execute the new processes. The selected development areas formed the basis for the second GDSS meeting, as they were considered essential for Eksote to be able to achieve their strategic objectives.

**Figure 5:**
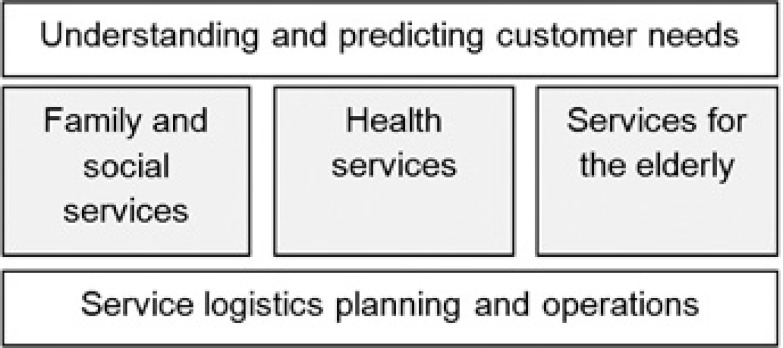
The main areas for improvement actions.

The first GDSS meeting resulted in tens of potential improvement actions to be carried out to overcome the identified challenges. Due to the large number of potential actions, a decision was made to focus on refining the actions and on selecting the most important ones for the Eksote action plan in the second GDSS meeting. In order to facilitate this process, the potential actions were divided into five main groups as shown in Figure 5. The main idea was that two of the action groups, i.e. understanding and predicting customer needs and service logistics planning and operations, were cross-functional and thus necessitated co-operation between different organisational units. The three remaining action groups were then specific to a certain organisational unit. The most important actions identified in the first GDSS meeting were entered into the corresponding groups and the template was then sent to the participants with the request to analyse and revise the content as preparation for the second GDSS meeting.

### Phase 3

The objective of the second GDSS meeting was to agree on the development projects for the roadmap leading Eksote towards an integrated organisation. The second GDSS meeting consisted of three main steps: (1) the potential development projects were identified and prioritised, (2) the expected benefits of the projects were estimated, and (3) the easiness of implementation of the projects was estimated. Thus, the second GDSS meeting resulted in a consensus decision on the development projects needed in the organisation, and a thorough analysis of the projects in terms of benefits and costs.

The structure of the second GDSS meeting is presented in Table 3. The main objective of the second GDSS meeting was to select a portfolio of improvement projects based on two criteria: expected benefits and easiness of implementation.

**Figure 6:**
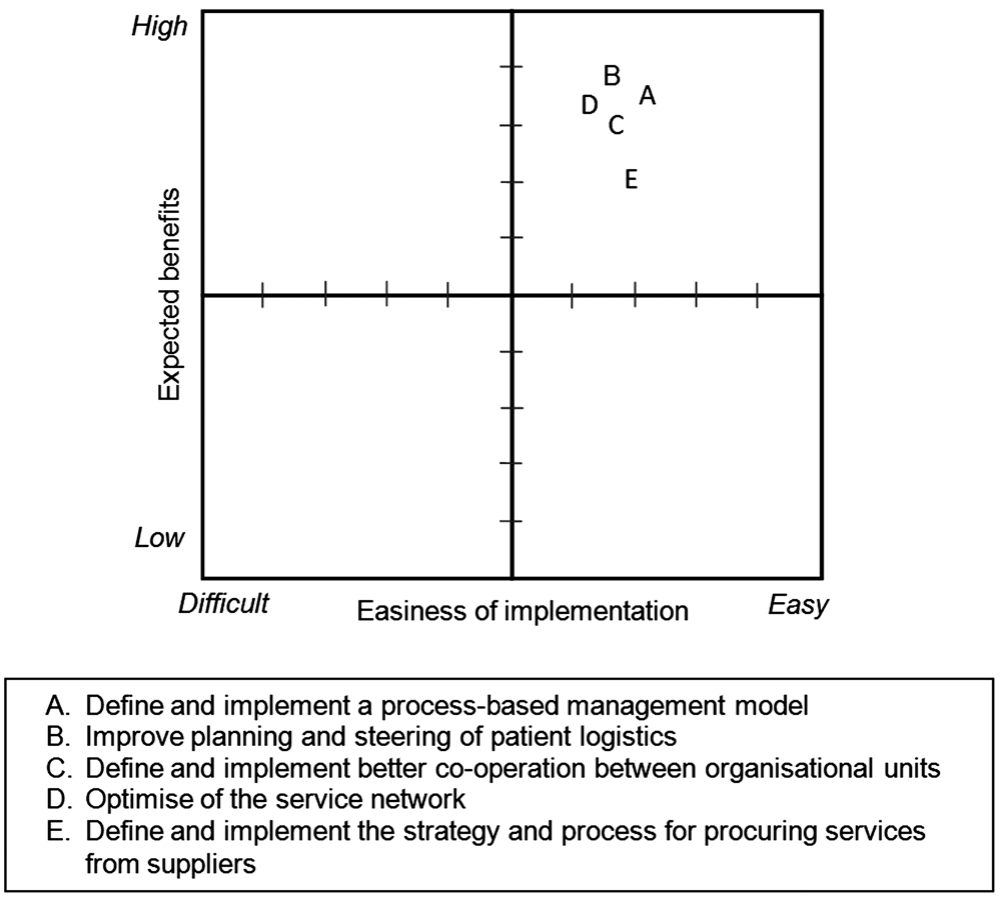
Analysis of the projects with regard to “Service logistics planning and operations”.

In the meeting, 20 improvement projects in the five predefined areas (Figure 5) were selected for the analysis, where each project was given a rating between 1 and 10 with regard to the two criteria. The results of the analyses were summarised to matrices in order to visualise the end result. An example of the positioning of the projects in the area “Service logistics planning and operations” is shown in Figure 6. The matrix shows that all the five projects in this area were expected to yield high benefits, and the implementation efforts were expected to be medium easy.

### Phase 4

The final phase was executed by a sub-team of the meeting participants. The team utilised the output of the second GDSS meeting to analyse and position the selected development projects in terms of estimated benefits and estimated easiness of implementation. On the basis of the in-depth analysis, the team summarised the results into a roadmap which showed the proposed sequence of executing the development projects. The roadmap covered a time span of three years into the future, and it created the foundation for the transition process to an integrated organisation.

**Figure 7:**
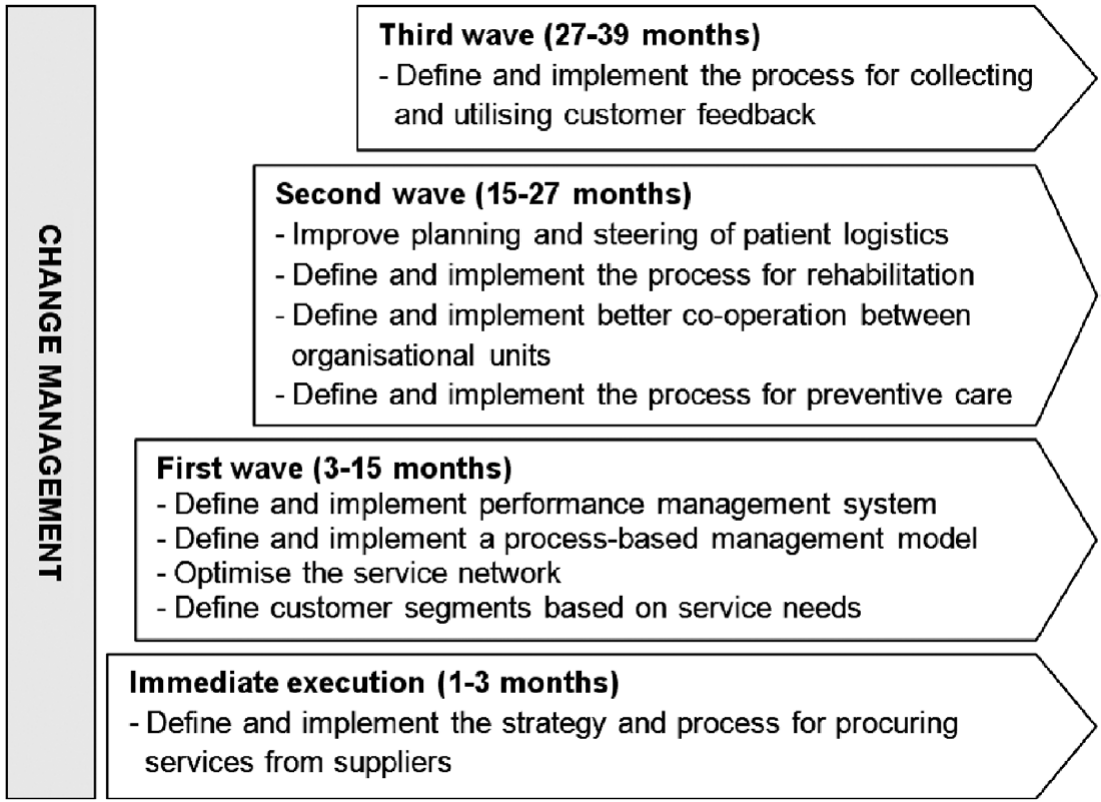
Eksote development roadmap.

The roadmap was prepared by deciding the sequence of execution for the projects. The execution sequence was decided on the basis of the matrices (see e.g. Figure 6) with the principle that the projects with highest expected benefits and the easiest implementation efforts would be implemented first. The roadmap showed the way forward for Eksote to achieve their strategic objectives in the most effective way. As shown in Figure 7, the roadmap consisted of different waves of actions. The implementation of two action waves had already been started, and the rest of the actions were divided into waves covering a time span of 2 years. Change management was identified as an ongoing effort needed to support the execution of the action waves. Furthermore, the roadmap was complemented by a detailed execution plan for each project inside the action waves. The execution plan described the steps to be taken, the responsible persons, detailed schedules, and prerequisites for execution. The roadmap was then communicated widely to the organisation, and the implementation was started.

## The advantages and challenges of the GDSS framework

Meeting satisfaction is an important measure of collaboration technology effectiveness, because unless the use of technology produces an increase in meeting satisfaction, it is unlikely that the users will seek to adopt the technology. In the Eksote case the participants experienced the designed collaboration process generally as effective and useful. According to the comments and the authors’ experiences, the participants saw the focused discussion on the important ideas and particularly on the major opinion differences very useful. The use of the GDSS process was seen to lead to goal-oriented, efficient group work, in which the focus was strictly on the issues at hand. The developed process was seen as time-efficient, bringing apparently significant time savings in the group work.

On the basis of the case experiences, it seems that the GDSS process can facilitate collaboration with different units and make the meetings more effective and fruitful. The process seems to help obtain in-depth understanding of development needs and requirements in a large organisational change. The case experiences also indicated that features that are likely to improve the development process are the ability to collect a large amount of detailed information and to analyse and evaluate the collected information with the formed development team with participants from different units.

It seems that the quality and usability of the results depend on the groups’ expertise on the subject. The meeting process supports group work but it does not replace the need of expertise and knowledge of the participants. Therefore it was very important to select people who knew about the challenges at Eksote and who had the needed authority for the implementation of decisions in the organisation.

The participants gave very positive comments about the meetings and the GDSS process. For instance, the following comments were noted down: “we got a lot of results in a short time”, “a good and effective way of working”, “the group size was adequate”, “the silent meeting stages gave an opportunity to consider the tasks profoundly” and “the quiet participants got their opinions heard”.

Overall, the comments of the participants were positive. In addition, Eksote has used GDSS in other development workshops after this case, which indicates high satisfaction with the process and the achieved results.

On the basis of the experiences from the case, the presented collaboration process can be seen as a suitable way to promote the generation, evaluation and selection of development projects. The process offers a systematic way to assess strategic plans, it facilitates the collection of opinions from different experts, and the logic behind the decisions made is documented well.

The developed process could be improved, however, by dividing the GDSS meetings into different parts, because going through many tasks in the same meeting is very laborious. For example the first GDSS meeting could have been arranged on two different days. Another uncertainty is the quality of the results; did the group find the most important and ‘right’ development projects? Anyway, the group was satisfied with the work done, and decisions for further actions were made.

## Conclusions

Integration of care is a challenge that organisations in the health and social services sector are currently facing. The development of integrated processes and organisations is a task that requires cross-functional commitment at all levels of the organisation. These types of collaboration processes are usually difficult, and thus advanced approaches are needed to increase their efficiency and effectiveness.

In this paper, a GDSS-supported collaboration process for defining projects to implement an integrated social and health care service strategy for the South Karelia District of Social and Health Services-organisation was introduced. The main result of the collaboration process was a detailed roadmap for the transition towards an integrated organisation. The process was divided into four phases, two of which were supported by a specific GDSS software.

The GDSS-based approach proved to be successful in supporting the creation of the roadmap. The utilised tool made it possible for the participants to discuss and analyse a large number of issues and tasks in a short period of time. Furthermore, anonymity of the participants in electronic discussion resulted in more open communication in the group. The GDSS-assisted collaboration process seemed to ensure a high level of commitment to the created plans in the group. Commitment, in turn, is essential to ensure that the created plans are implemented in practice.

According to the case experiences it seems likely that the developed process can also work in other strategy implementation problems of similar kind. There are several characteristics in the developed collaboration process that are likely to promote the strategy implementation. Identified general characteristics are for example: careful preplanning of the collaboration, systematic handling of meeting phases, effective facilitation of a group of experts from different units, possibility for equal participation, automatic documentation of the input, and possibility to work anonymously.

## Figures and Tables

**Table 1.**
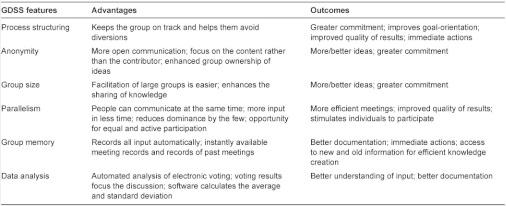
Benefits of the GDSS [12, 13]

**Table 2.**
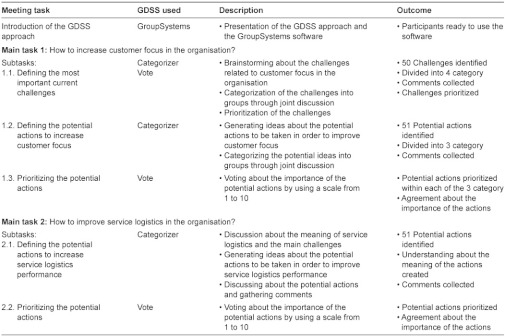
Process chart of the structure of the first GDSS meeting

**Table 3.**
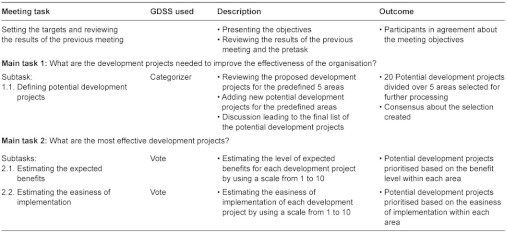
Process chart of the structure of the second GDSS meeting

## Footnotes

1 Chief executive officer, associate chief physician, chief administrative physician, acting director of the elderly services, development director, home care director, mental care director, primary care director, acute care director, long time care director, family and social services director, project manager, process engineers, social service director, social workers, nurses, physiotherapists, and family doctors.

## Reviewers

**Leonid Chechurin**, Professor, Head of Innovation Theory Department, St. Petersburg State Polytechnic University, Russia

**Teija Hammar**, RN, DSc (health economics), Senior Researcher, National Institute for Health and Welfare, Helsinki, Finland

**Sirkka Sinkkonen**, PhD, emerita professor in Health Services Management, Department of Health and Social Management, University of Eastern Finland (until 1.10.2010 University of Kuopio)

**Helena Taskinen**, PhD, Senior Researcher, University of Eastern Finland, Finland
